# Incidence of COVID-19 infection and its variation with demographic and clinical profile: lessons learned at a COVID-19 RT-PCR laboratory in Nagpur, India

**DOI:** 10.1099/acmi.0.000330

**Published:** 2022-03-04

**Authors:** Neeta Gade, Soumyabrata Nag, Meena Mishra, Sujiv Akkilagunta, Vishal Shete, Vijay Bidkar, Pooja Shendre, Divya Patil

**Affiliations:** ^1^​ Department of Microbiology, AIIMS, Nagpur, India; ^2^​ Department of Community Medicine, AIIMS, Nagpur, India; ^3^​ Department of ENT, AIIMS, Nagpur, India

**Keywords:** COVID-19, demography, inconclusive, RT-PCR

## Abstract

**Introduction.** The coronavirus disease 2019 (COVID-19) pandemic emerged as a global health crisis in 2020. The first case in India was reported on 30 January 2020 and the disease spread throughout the country within months. Old persons, immunocompromised patients and persons with co-morbidities, especially of the respiratory system, have a more severe and often fatal outcome to the disease. In this study we have analysed the socio-demographic trend of the COVID-19 outbreak in Nagpur and adjoining districts.

**Methods.** The study was conducted from April to December 2020. Nasopharyngeal and oropharyngeal swabs collected from suspected cases of COVID-19 were tested using reverse-transcription polymerase chain reaction (RT-PCR) at a diagnostic molecular laboratory at a tertiary care hospital in central India. Patient-related data on demographic profile and indication for testing were obtained from laboratory requisition forms. The results of the inconclusive repeat samples were also noted. The data were analysed using SPSS v24.0.

**Results.** A total of 46 898 samples were received from April to December 2020, of which 41 410 were included in the study; 90.6 % of samples belonged to adults and 9.4 % belonged to children. The overall positivity rate in the samples was 19.3 %, although it varied over the period. The yield was significantly high in the elderly age group (25.5 %) and symptomatic patients (22.6 %). On repeat testing of patients whose first test was inconclusive, 17.1% were positive. There was a steady increase of both the number of tests and the rate of positivity in the initial period of the study, followed by a sharp decline.

**Conclusion.** We can conclude that rigorous contact tracing and COVID-appropriate behaviour (wearing a mask, social distancing and hand hygiene) are required to break the chain of transmission. Elderly people are more susceptible to infection and should follow stringent precautions. It is also important to perform repeat testing of those individuals whose tests are inconclusive with fresh samples so that no positive cases are missed. Understanding of demographics is crucial for better management of this crisis and proper allocation of resources.

## Introduction

Coronavirus disease 2019 (COVID-19) is an infectious disease caused by the newly discovered severe acute respiratory syndrome coronavirus 2 (SARS-CoV-2). The COVID-19 outbreak began in Wuhan, Hubei Province, PR China in December 2019 [1]. The first case of COVID-19 in India, which originated from PR China, was reported on 30 January 2020 [2]. Later, on 11 March 2020, the World Health Organization (WHO) characterized the COVID-19 outbreak as a pandemic. India currently has the largest number of confirmed cases in Asia and the second highest number of confirmed cases in the world [3].

In humans, several coronaviruses are known to cause respiratory infections, ranging from the common cold to more severe diseases, such as Middle East respiratory syndrome (MERS) and severe acute respiratory syndrome (SARS). SARS-CoV-2 is an enveloped positive-stranded RNA virus whose genome contains ~30 000 nucleotides and 15 genes. The whole-genome sequence of SARS-CoV-2 shows that its closest relationship is with the bat SARS-like coronavirus strain BatCov RaTG13, with 96 % identity [4].

The disease spreads primarily from person to person through small droplets and aerosols [5, 6]. The incubation period is ~5–6 days (range 1–14 days). It can be transmitted during the asymptomatic incubation phase (in ~50–60 % of cases) and for up to 2 weeks after the onset of symptoms. Clinical presentation varies from asymptomatic or mild illness to severe or fatal disease; deterioration can occur rapidly, often during the second week of illness [7]. Old persons, immunocompromised patients and persons with co-morbidities, especially of the respiratory system, have a more severe and often fatal outcome to the disease. Air pollution, average age of population, population density and economic factors also play a role in determining the severity of the outbreak in a particular geographical location [8].

In this study we have analysed the socio-demographic trend of the COVID-19 outbreak in Nagpur, Maharashtra, India and its adjoining districts of Vidarbha region of Maharashtra. Further, the correlation of the category of patient with COVID-19 positivity was studied, along with challenges in laboratory diagnosis. The secondary objective was to assess the outcome of inconclusive samples on repeat testing.

## Methods

### Study setting

The study was conducted in an Indian Council of Medical Research (ICMR)-approved diagnostic molecular laboratory, AIIMS, Nagpur, Maharashtra, India. Nagpur is one of the heavy-burden districts in Maharashtra. The laboratory received samples from all the districts in the Vidarbha region of Maharashtra.

### Study type

The study is a record-based analytical study.

### Study period

Samples received at the designated COVID-19 laboratory between April and December 2020 were included in the analysis.

### Sample collection and testing

Upper respiratory tract specimens (nasopharyngeal and oropharyngeal swabs) were collected from suspected cases of COVID-19 at various centres in the Vidarbha region of Maharashtra, as per ICMR guidelines [9–11]. In order to optimize resources, the ICMR suggested indications for testing based on travel history, contact history, signs and symptoms and high-risk groups. These indications have been revised by the ICMR from time to time, based on epidemiological data [9–11]. The testing of samples was performed at the diagnostic molecular laboratory, AIIMS, Nagpur. The demographic details of the patients were also noted. The samples were accepted for testing with the cold chain being maintained in viral transport medium. Samples were processed and RNA was extracted using column-based and automated RNA extraction kits. Reverse-transcription polymerase chain reaction (RT-PCR) assays were performed for the detection of the E (envelope), N (nucleocapsid), RdRp (RNA-dependent RNA polymerase), and ORF1ab genes with the RTPCR kits provided by the government. Each kit was tested for two genes among the ones mentioned above. Cut-off *C*_T_ values were used to determine the sample positivity as per the manufacturer’s instructions. A sample was only considered positive if the *C*_T_ values of both genes were below the cut-off. If the *C*_T_ value of any came below the cut-off (<35) and the other came above it, the result was considered inconclusive. On repeat sampling of these inconclusive samples, if both the genes were detected at a *C*_T_ value <35, then it was considered positive.

### Data collection

Patient-related data on demographic profile and indication for testing were obtained using specimen referral forms received in the laboratory. Being a record-based study, waiver of consent was obtained from the institutional ethics committee. The samples were excluded if data on one or more of the following parameters were missing – age, gender and indication for testing. Children were considered to be those below 18 years of age and were divided into two subgroups (0 to 9 years and 10 to <18 years). During the analysis, the data were recorded using uniform codes for testing criteria for samples received across the entire duration of the study. The new criteria were also incorporated [9–11]. In some laboratory forms, the specific testing criterion was not marked, but the indication of testing was noted in descriptive form. Such data were also recorded.

For the secondary objective, the repeat (paired) samples were identified based on specimen referral form (SRF) ID, demographics and mobile number. Based on the paired samples identified, the result of the inconclusive samples on repeat testing was analysed. The initial treatment guidelines mandated that the patients should test negative before discharge from hospital. The results of the paired follow-up samples of COVID-19-positive patients were analysed separately.

### Statistical analysis

The data were cleaned in MS Excel and analysed using SPSS v24.0. Quantitative variables were summarized as median (range) and categorical variables were summarized as proportions. Positivity rate or yield was reported for subgroups based on demography and testing criteria. The chi-square test was used to find the association of demographic and clinical profile with positivity rate. The positivity rate trend for all samples tested at the laboratory and for district-based subgroups was reported.

## Results

A total of 46 898 samples were received from April to December 2020, of which 41 410 were included in the study; 5488 samples were excluded from primary analysis because of duplication (*n*=2588), improper sampling (*n*=910), inconclusive (*n*=1310) and incomplete data (*n*=680). For the secondary objective, the inconclusive samples were included. Of these, 427 patients (854 paired samples) who came for repeat testing were identified based on multiple identifiers, including mobile number and SRF ID.

Among the samples that were included for the study, 37 531 (90.6 %) belonged to adults, i.e. those ≥18 years, and only 3879 (9.4 %) belonged to children. The socio-demographic distribution of the population is presented in Table 1. In the study sample, more than half (62.3 %) were male. Most of the patients were young adults belonging to the age group of 18–29 years (29.8 %), followed by the age group of 30–39 years (27.3 %). Only 8.5 % of the patients were elderly (60 years and above). Majority of the patients came from Nagpur district (62.9 %), followed by Chandrapur (18.7 %).

**Table 1.** Socio-demographic distribution of the study sample and yield of RT-PCR (*n*=41 410)

Sociodemographic characteristic	Categories	Total cases	Yield/ Positivity rate	p-value
**Children (*n*=3879)**	0 to 9	1576	22.9 %	<0.001 P-for trend – 0.007
	10 to <18	2304	20.1 %	
**Adults (*n*=37531)**	18 to 39	21 398	18.2 %	
	40 to 59	12 964	18.9 %	
	60 and above	3168	25.5 %	
**Gender**	Female	15 611	20.1 %	0.001
	Male	25 788	18.7 %	
	Transgender	11	45.5 %	
**District of Residence**	Nagpur	26 053	23.7 %	<0.001
	Chandrapur	7736	11.1 %	
	Gadchiroli	4336	11.5 %	
	Yavatmal	1038	10.1 %	
	Buldhana	574	19.5 %	
	Others	1673	15.3 %	
**Clinical Profile**	Asymptomatic	31 849	18.3 %	<0.001
	Symptomatic	9557	22.6 %	

With respect to ICMR category, almost half of the patients were tested (46.7 %) because they were asymptomatic but in direct and high-risk contact with a confirmed case of COVID-19. The overall positivity rate in our samples was 19.3 %, although it varied over the period. The association of COVID-19 positivity rate with demographic parameters is presented in Table 1. The yield was significantly high in the elderly age group (25.5 %), followed by the 0–9 years age group (22.9 %). There was an increasing trend of positivity among adults with increasing age. The positivity rate was high among female COVID suspects (20.1 %) when compared to male suspects (18.7 %) (*P*=0.001). Subgroup analysis was performed to assess the gender variation in positivity. When assessed among various subgroups, this gender variation was not significant. Among healthcare workers, there was no significant difference in positivity across gender (male vs female – 19.8 % vs 22.6 %; *P*=0.1). Similarly, gender variation was not significant across all the major districts in the study except for Chandrapur (male vs female – 14.2 % vs 9.4 %; *P*=0.1). Symptomatic patients had significantly high positivity rate (22.6 %) when compared to that of asymptomatic patients (18.3 %) (*P*<0.001).

**Fig. 1.**
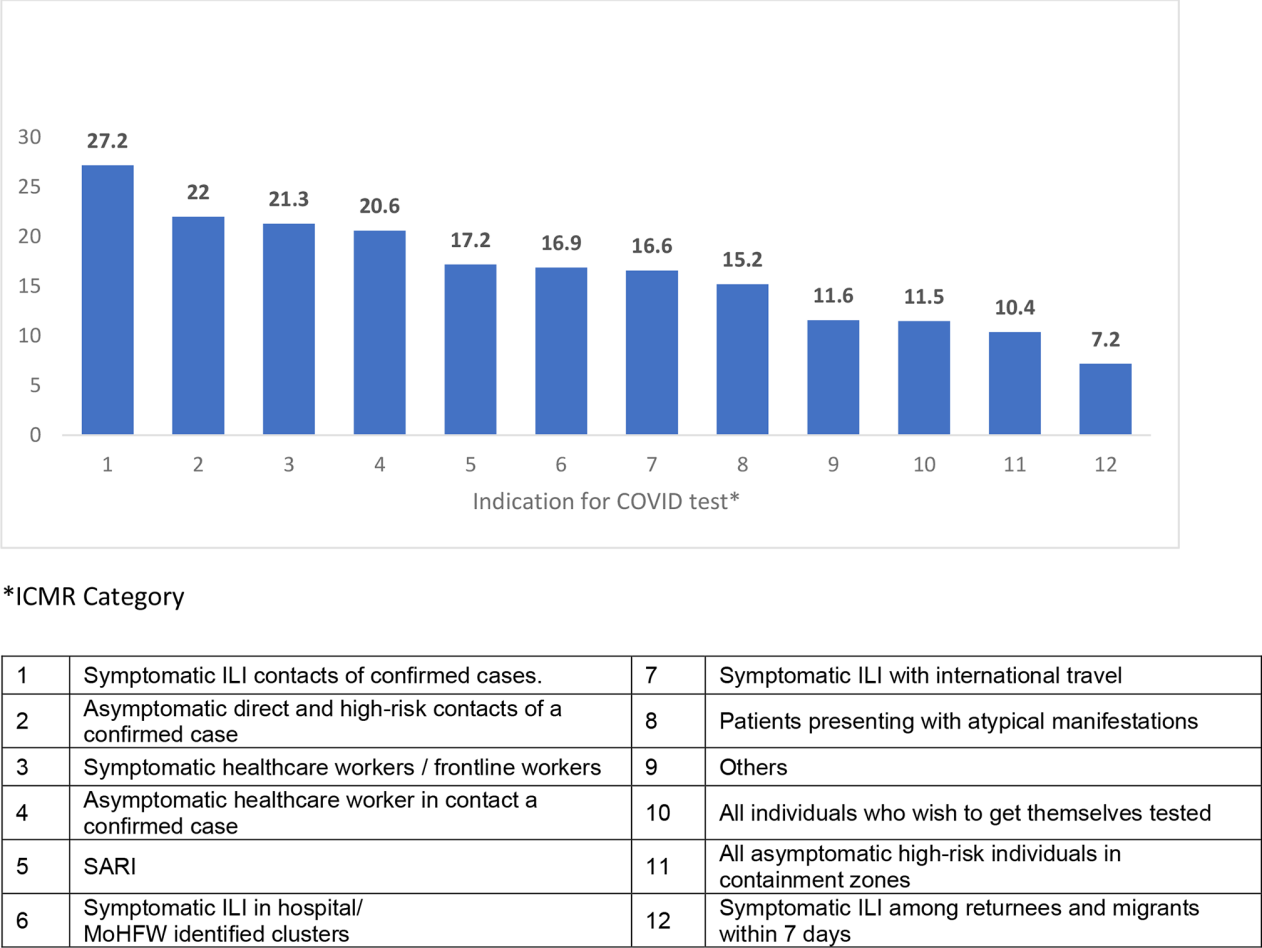
Positivity rate based on indication for COVID test as per ICMR guidelines.

When analysed based on the indication for COVID-19 test, the highest yield was observed among symptomatic contacts of laboratory-confirmed cases (27.2 %), followed by asymptomatic contacts (22 %). (Fig. 1) Among healthcare workers, however, the positivity rate did not vary significantly between symptomatic (21.3 %) and asymptomatic individuals (20.6 %). The overall positivity rate in severe acute respiratory illness (SARI) cases was 17.2 %.

**Fig. 2.**
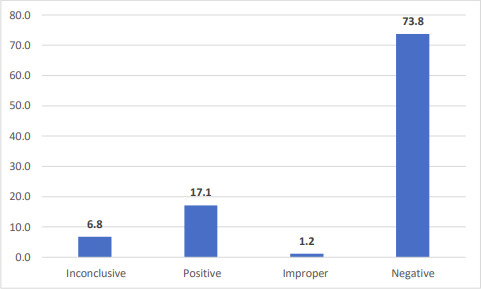
Distribution of RT-PCR result among inconclusive samples (*n*=427 paired/follow-up samples).

Of the 427 patients for whom repeat samples were taken because the first test result was inconclusive, 17.1 % were positive on repeat testing and 6.8 % remained inconclusive. The majority of samples turned out to be negative (73.8 %) (Fig. 2).

A total of 40 patients were tested during follow-up. Nineteen of them (47.5 %) remained positive after a median follow-up of 15 days.

A subgroup analysis was performed for children included in the sample. In the paediatric population, most cases belonged to the age group 10–14 years (33.8 %), followed by 5–9 years (22.4 %). Most of them (58.5 %) were tested because they were in direct and high-risk contact with a confirmed case of COVID-19. The overall positivity rate among children (21.3 %) was slightly high when compared to adults (19.1 %). In children, the positivity rate was highest in the 5–9 years (24.3 %) age group, followed by the 1–4 years age group (22.1 %). A significant positive association was also observed between female gender and COVID-19 positivity in children. However, in children this difference was more pronounced [female (26.3 %) vs male (18.1 %)]. On the other hand, asymptomatic children had high positivity (21.5%) when compared to symptomatic (20.5%), but this was statistically insignificant (*P* value=0.56).

**Fig. 3.**
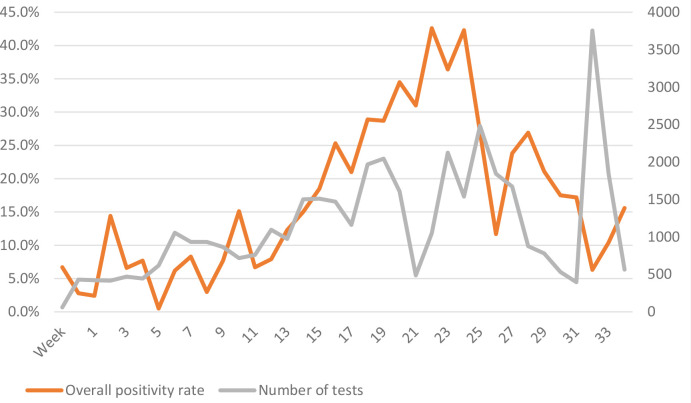
Weekly trend of RT-PCR test volume and COVID-19 positivity rate in the designated laboratory.

**Fig. 4.**
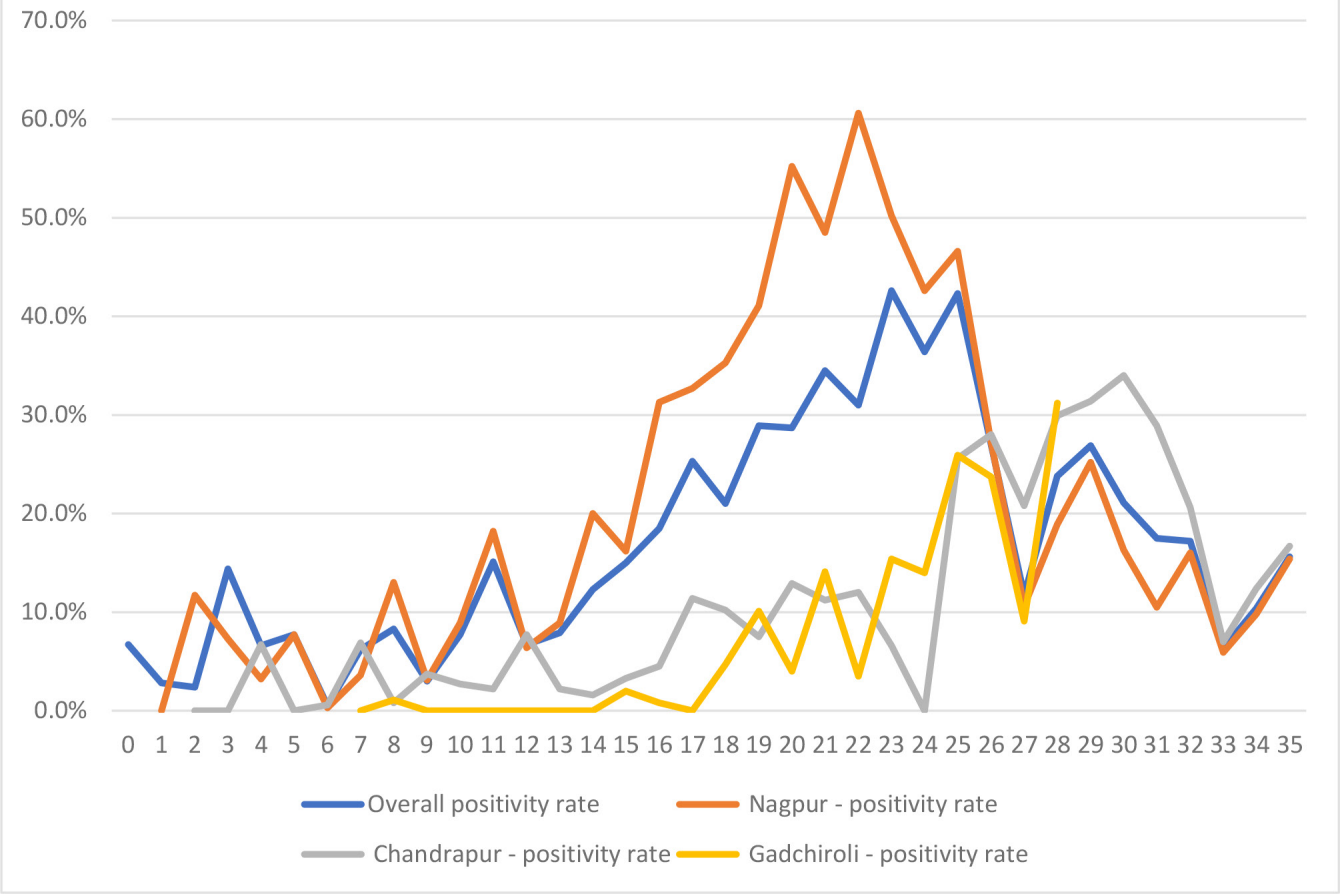
Weekly trend of COVID-19 positivity rate among samples tested from selected districts in Vidarbha region.

The trend of the COVID-19 positivity of samples received in the laboratory across the study period and three major burden districts – Nagpur, Chandrapur and Gadchiroli – is presented in Figs 3 and 4. There was a steady increase in both the number of tests and the positivity rate in the initial period of the study, followed by a sharp decline (Fig. 3). The increase in positivity rate was, however, without an increase in the volume of tests. Among different districts, the peak was staggered across the districts – the peak in Nagpur was followed by a smaller peak in other districts.

## Discussion

The present study is an attempt to gain an insight into the relation between the COVID-19 outbreak and demographic and geographical factors. This pathogen spreads through the structure of social contacts, which in turn varies with age; hence, it is necessary to analyse age distribution and social customs in a population to better analyse the spread of the contagion [12]. The present study showed high COVID-19 positivity among female suspected COVID-19 patients. The positivity rate was high among the extremes of the age groups – children and the elderly. These findings could be a surrogate marker of demographic risk factors in the general population [13–16].

In the present study it was observed that during the first wave of the COVID-19 pandemic there was an initial steady rise in the number of tests as well as positivity, followed by a sharp decline, in Nagpur. The graph was similar to that for India as a whole and also other countries.

The were significantly more male than female patients in our study, but the positivity rate was significantly higher in females. A study from southern India by Laxminarayan *et al.* during a similar time period (May to October 2020) reported an overall positivity rate of 3.6 %, with a slightly higher positivity rate among males. This is in contrast to our observations, and this variation could be attributed to the difference in geographical location, sample size and relative burden of COVID-19 infection [17].

In our study, we found that mostly young adults were being tested, with the most common reason being contact with a confirmed case of COVID-19. This was probably because they are more likely to be students/employed and are more likely to have more interactions with other people despite lockdown in the country. A study from Japan by Mizumoto *et al.* shows that children are less likely to be diagnosed as cases and also the risk of disease given exposure among children is low. Both the overall risk and the conditional risk of disease are highest among adults aged >50 years [18]. In our study, we found that <10 % of those tested were children, making them less likely to be diagnosed if not symptomatic. We observed that asymptomatic children had high positivity (21.5 %) when compared to symptomatic (20.5 %), which is in concordance with the studies who reported that children appear to be more likely to have asymptomatic infection than adults [19].

Any person coming into contact with a confirmed case of COVID-19 and those showing symptoms of COVID-19 should be tested immediately. The yield of positivity in such cases is much higher than the other categories, as shown in Fig. 2. This is in line with the WHO recommendation for testing [20].

Molecular targets for SARS-CoV2 RT-PCR detect at least two genes of the virus. The detected genes can be E (envelope), N (nucleocapsid), S (spike) or RdRP (RNA-dependent RNA polymerase), depending on the kit used. An inconclusive result is given when only one of the two genes is detected. In this study 1310 samples were inconclusive after the first test. This can be because of a number of reasons: (1) another beta-coronavirus infection, (2) non-specific binding of primer probes during PCR cycle, (3) the different sensitivity of different viral genes at low viral load (early infection), (4) a problem in RNA extraction and (5) poor sample quality.

Repeat sampling and testing is advised for inconclusive results (i.e. only the E or N gene was detected), as it may be a sign of early infection. In our study, out of the 427 repeat samples from patients whose first samples were reported to be inconclusive, 17.1 % turned out to be positive. Thus, repeat testing should be performed despite the fact that it increases the turnaround time and adds cost to the healthcare system. Diagnostic stewardship (correct time and method to collect samples) can be applied to decrease the number of inconclusive results, which in turn will conserve laboratory supplies [21].

## Conclusion

The authors present their experience regarding the demographics of the first wave of the COVID-19 pandemic in Nagpur, Maharashtra, India and its adjoining districts in the Vidarbha region, Maharashtra. We can conclude that rigorous contact tracing and COVID-appropriate behaviour (wearing a mask, social distancing and hand hygiene) are required to break the chain of transmission. Elderly people are more susceptible to infection and should follow stringent precautions. We observed that the first wave of the pandemic mainly affected the elderly population. The majority of the people were tested after contact with a confirmed case, although they were asymptomatic. A considerable positivity rate in them suggests that rigorous contact tracing is required break the chain of transmission. It is also important to perform repeat testing for those individuals with inconclusive tests, using fresh samples, so that no positive cases are missed. Nagpur is currently facing the second wave of the COVID-19 pandemic. Understanding the demographics is crucial for better management of this crisis and proper allocation of resources.
